# Anxiety, Worry, Life Satisfaction and Coping During the Acute VS Prolonged Pandemic Stress: Evidence From a Repeated Cross-Sectional Study

**DOI:** 10.3389/ijph.2022.1604650

**Published:** 2022-06-01

**Authors:** Ia Shekriladze, Nino Javakhishvili, Nino Butsashvili, Maka Lortkipanidze

**Affiliations:** School of Arts and Sciences, Ilia State University, Tbilisi, Georgia

**Keywords:** anxiety, pandemic, worry, life satisfaction, coping

## Abstract

**Objectives:** The COVID-19 pandemic remains a continuous stressor worldwide. Our study aimed at comparing the data of waves from two lockdowns in Georgia, one in the acute stress phase (May 2020) and the other in the prolonged stress phase (December 2020).

**Methods:** In total, 750 and 716 individuals participated in the study with a repeated cross-sectional design. Sample equivalence was reached *via* controlling demographic variables. Anxiety, COVID-19 worry, and life satisfaction were measured along with coping behaviors and four coping styles—information-accessing/processing and action-planning (two problem-focused coping styles), and passive-submissive and avoidant (two emotion-focused coping styles).

**Results:** As pandemic prolonged, mental health indicators worsened, the action-planning style and behavioral coping decreased, while the information-accessing/processing style increased. The link between the COVID-19 worry and the action-planning coping style was strong in the acute stage and dissapeared in the prolonged stage. The individual context, namely, a history of coronavirus in the household, accounted for lower protective behaviors and higher information seeking in the prolonged phase.

**Conclusion:** The findings highlighted the importance of timing and general and individual contexts in coping with the pandemic.

## Introduction

The novel coronavirus (COVID-19) outbreak, having reached pandemic level in March 2020, still remains a global challenge. Evidence suggests that public mental health is considerably affected during lockdowns caused by outbreaks of infectious diseases, as the pressing need for adjustment to multiple changes disrupt individuals’ sense of stability and elevate their stress levels [1, 2]. The COVID-19 pandemic and subsequent public health measures affected population wellbeing, as studies report increased levels of anxiety, depression, and distress among diverse groups [3, 4], reiterating the critical need for promoting resilience during the global crisis.

In Georgia, the first case of the virus was identified in February 2020 and the first lockdown was implemented in March–May 2020, followed by stronger waves of the virus and the second lockdown in November, 2020. Both lockdowns included a 11 p.m.–6 a.m. curfew, closing of schools/offices, and restriction of travel. While by the end of the spring lockdown, Georgia had only 723 confirmed cases of virus and 12 deaths, during the second lockdown the spread of the infection reached 5450 new daily cases and 53 daily deaths. Vaccination was not launched until March, 2021. The collective stress triggered anxiety and worry about COVID-19 among public even at the early stage of the pandemic [5, 6].

Being an integral part of human life, stress has generated considerable interest in the fields of medicine and psychology: its impact on individuals’ health and wellbeing has been studied for decades. Back in the 1970s, Selye described a common response pattern to stress called the general adaptation syndrome that comprises three phases: 1) the alarm stage characterized by an increased defensive reaction, 2) the resistance stage characterized by adaptation to the stressor, and 3) the exhaustion stage, associated with the greatest damage, during which the organism’s capacity of adapting to the ongoing stressor is exhausted [7, 8]. This pattern illustrates the differences between the acute and chronic stress, pinpointing detrimental effects of prolonged exposure to a stressor.

Later, Lazarus and Folkman [9] defined psychological stress as a transaction between an individual and their environment, in which demands exceed the available coping resources thereby qualified as jeopardizing for wellbeing. Coping has been defined as the use of cognitive and behavioral strategies to manage the demands of situations that are appraised as taxing [10, 11].

The transactional theory of stress and coping emphasized that an individual’s capacities and resources largely determined their responses to a stressor, and distinguished between the primary and secondary control. According to this model, problem-focused coping (primary control) is favored when there are resources available to handle the stressor, while emotion-focused coping (secondary control) is appropriate when the stressor is uncontrollable [9]. While problem-focused coping implies dealing with the disturbing person-environment relationship by attempting to change the environment to reduce the stressor, emotion-focused coping encompasses modifying one’s reaction or interpretation towards the stressful relationship.

When the spread of COVID-19 reached its pandemic level, it emerged as an acute stressor for populations at large: increased levels of anxiety and alarm were reported by multiple studies [12–15]. With time, the pandemic progressed into a continuous global stressor. A cross-sectional population study examining trends of perceived stress, anxiety and worry at four different points of the pandemic showed persistent levels of anxiety and worry [16]. Another two-phase multi-country study identified an increase in depression and anxiety indicators in eight different countries [17].

The pandemic and the associated preventive measures affected the quality of life of individuals. Lower levels of life satisfaction were linked with frustration, depression, fear of COVID-19 in studies conducted in Europe and Asia [18–20]. Higher life satisfaction was linked with lower social isolation [21, 22] and a sense of having access to information [21].

During prolonged collective crisis much depends on individual capacity for coping. While some studies linked problem-focused coping with healthier functioning and emotion-focused coping with less favorable outcomes [23], others suggested the advantage of emotion-focused coping when the stressor was uncontrollable [24]. Research on pandemics generated mixed evidence: on one hand, emotion-focused coping was associated with greater anxiety and fear of COVID-19 [25, 26], well-being was positively linked with problem-focused coping and negatively correlated with disengagement coping [27]; on the other hand, both rational and affective coping were linked with greater levels of anxiety [28], and psychological distress significantly predicted both ways of coping [29].

Evidence from spring, 2020 showed that anxiety predicted affective coping and negatively predicted rational coping, while COVID-19 worry predicted both affective and rational coping. These findings suggested that a threat-oriented worry tended to enhance all types of coping, whereas anxiety acted as a barrier to problem-focused coping [6].

Putting pandemic into the framework of general adaptation syndrome suggests that spring, 2020 represented an acute stage of stress for the majority of populations, corresponding to the alarm reaction phase, while autumn 2020 represented the prolonged stage of stress associated with the exhaustion phase. Thus, the response patterns may vary accordingly. Furthermore, based on the transactional theory of stress and coping, the extents of primary and secondary control may vary depending on the local epidemiological situation and available resources.

Our study, thus, aimed at examining population response to the pandemic at two different points–the acute (May 2020 lockdown) and prolonged (December 2020 lockdown) stress phases–in order to explore patterns and variations in responses to stress. We examined the levels of state anxiety, COVID-19 worry, and life satisfaction as well as coping styles to explore whether two different pandemic-related lockdowns provided a backdrop for significant changes in emotional and behavioral responses. Individual/household demographics were also examined in respect of the major variables.

We expected an increase in anxiety and COVID-19 worry and a decrease in life satisfaction and coping behaviors over time as a result of prolongation of the pandemic. Following Selye’s theory and the transactional theory of stress and coping, we anticipated higher rates of problem-focused and behavioral coping during the acute phase and an increase in emotion-focused coping during the prolonged phase.

## Methods

### Design

A repeated cross-sectional design was used to identify differences between two phases. The study was conducted in accordance with the Declaration of Helsinki. The ethics approval (R/182-20) was obtained from the Ilia State University’s Research Ethics Committee.

#### Participants and Procedure

The study was conducted *via* electronic survey containing several self-report questionnaires. The selection method was convenience sampling. The study link was extensively distributed through social media and other electronic means. Eligible participants consisted of Georgian speaking individuals aged 18 or older. To increase participation of diverse groups, a booster was used. The survey was anonymous to encourage participation and frankness. All instruments listed below were included in the survey links of both phases.

The first wave of the study was completed in May, 2020 during the first lockdown, whereas the second wave was completed in early December, 2020 during the second lockdown. To achieve homogeneity of the samples, consistency was kept between the selection methods of data collection.

In total, 1581 Georgian adults aged 18–88 participated in both phases of the study: 849 in the first phase and 732 in the second. The mean age of the first wave equaled 37.50 (*SD* = 13.37) and 80% of the sample were women. In the second wave, the mean age was 34 (*SD* = 13.1) and women comprised 76% of the sample. Because certain divergence in the sample compositions was detected, we removed 99 participants from the first sample and 16 participants from the second sample and ended up with sample sizes of 750 and 716 respectively. More detailed information about samples is presented in the results section.

### Measures

The following instruments were used to measure the main variables, each accompanied by a 5-point Likert Scale from 1–“fully disagree” to 5–“fully agree”.

The State Anxiety Inventory [30] is a 19-item (20 items in the original version) self-report questionnaire that measures a person’s current level of anxiety. The inventory was previously validated for the Georgian population [31]. Cronbach’s alpha amounted to 0.93 for the first wave and 0.92 for the second.

The COVID-19 Worry Scale is a 3-item self-report inventory that measures the overall concern with COVID-19. The scale was borrowed from a German study [32] and one item was added. It measured general worry about COVID-19, the fear of getting infected by COVID-19, and the fear of a family member contracting COVID-19 (added). The scale underwent Exploratory Factor Analysis (EFA) [6]. Cronbach’s alpha equaled to 0.77 (the first wave) and 0.75 (the second wave).

The Satisfaction with Life Scale [33] is a 5-item inventory measuring a person’s global judgment of satisfaction with life as a whole. The inventory was previously validated for the Georgian population [25]. Cronbach’s alphas amounted to 0.83 (the first wave) and 0.82 (the second wave).

The Ways of Coping Scale (Georgian version) is an 18-item self-report questionnaire measuring an individual’s problem-focused and emotion-focused coping styles in response to the pandemic on four sub-scales: 1) the Action-Planning subscale, 2) the Information-Accessing/Processing subscale, 3) the Passive-Submissive subscale, and 4) the Avoidant subscale. The first two sub-scales represent problem-focused coping styles, and the last two sub-scales - emotion-focused coping styles. Borrowed from a German study [32], the tool underwent Confirmatory Factor Analysis (CFA) and was subsequently modified into four sub-scales [6]. The German version of the instrument was a revised and adapted version of the Ways of Coping Questionnaire [34] comprising the Problem-Focused and Emotion-Focused Ways of Coping subscales. Cronbach’s alphas amounted to 0.78 (the first wave) and 0.85 (the second wave) for the action-planning subscale, 0.79 (the first wave) and 0.85 (the second wave) - for the information assessing/processing subscale, 0.69 (the first wave) and 0.72 (the second wave) - for the avoidant subscale, and 0.62 (the first wave) and 0.65 (the second wave) - for the passive-submissive subscale.

The Pandemic-Related Coping Behaviors Scale is a 4-item self-report inventory on Behavioral Dimensions of Coping (washing hands, cleaning stuff, keeping social distance, and avoiding public places) [32]. The scale featured behaviors equally appropriate for both lockdowns in order to ensure the comparability of the findings. EFA using principle components analysis with Varimax rotation yielded two factors. As calculating Cronbach’s alpha is not advised for two-item scales [35], inter-item correlations were done: Spearman-Brown coefficient r for regular coping behaviors was 0.75 (the first wave) and 0.83 (the second wave); Spearman-Brown coefficient r for pandemic-specific behaviors equaled 0.72 (the first wave) and 0.66 (the second wave), respectively.

The study gathered information on demographic and household variables including age, gender, marital status, employment status, the number of household members, the numbers of children and elderly in the household, presence of chronic illness, and history of coronavirus.

### Statistical Analysis

Data were analyzed using the statistical package IBM SPSS version 23.00. Consistency and reliability of the factor loadings were tested by Cronbach’s alpha, with values higher than 0.6 considered appropriate. Descriptive statistics were calculated for all the variables. Bivariate correlational analyses were performed using Pearson’s r coefficient. Mean scores of the main variables were compared between the two samples via MANOVA and MANCOVA. Probability level of *p* < 0.05 was used with some statistical tests of significance, while alpha of 0.05 was used for others.

## Results

### Preliminary Analysis Results

Removal of a number of participants from both samples yielded similar demographic profiles. To ensure the samples were homogenous for comparison, we conducted tests for homogeneity of variance for all parameters. We used Levene’s Test for Equality of Variances to check age homogeneity across two samples, which was non-significant *F* = 2.49, *p* > 0.05. Homogeneity of all other variables was checked by comparing their distributions: for gender–χ^2^ (1) = 2.38, *p* > 0.05; for employment status–χ^2^ (7) = 11.46, *p* > 0.05; for marital status–χ^2^ (4) = 8.20, *p* > 0.05; for having elderly in a family–χ^2^ (6) = 7.75, *p* > 0.05; for having children in a family–χ^2^ (12) = 14.51, *p* > 0.05; and, finally, for having chronic illness χ^2^ (3) = 2.11, *p* > 0.05; and having family members with chronic illness–χ^2^ (7) = 9.85, *p* > 0.05. Since the homogeneity of variances across two samples was demonstrated, we gained firm grounds for proceeding with the comparisons of the variables of the two waves (see Table 1).

**TABLE 1:** Sample demographic and household characteristics (Mental Well-being and Coping during the COVID-19 Pandemic, Georgia, 2020).

		Wave I N = 750	Wave II N = 716	χ^2^	*p*
		%	%		
Gender	Female	79.20	75.80	2.38	0.069
	Male	20.80	24.20		
Marital Status	Single	44.30	51.7	8.20	0.085
	Married	39.90	34.50		
	Divorced	9.60	8.50		
	Widow	3.70	3.40		
Employment	Employed	72.50	70.90	11.46	0.120
	Unemployed	7.00	8.70		
	Student	15.90	15.40		
	Retiredact	2.00	0.80		
Having chronic illness	yes	7.10	7.50	2.11	0.550
	No	92.90	92.50		
Number of household members	0	7.30	8.70	11.26	0.187
	1	16.90	17.30		
	2	19.60	17.70		
	3	25.60	23.50		
	>3	30.50	32.80		
Number of children in household	0	52.80	58.50	14.52	0.269
	1	24.00	21.40		
	2	15.90	13.10		
	3	4.70	4.50		
	>3	2.70	2.50		
Number of elderly in household	0	70.10	68.70	7.75	0.257
	1	23.70	23.40		
	2	5.70	6.30		
	3	0.40	0.80		
	>3	0.00	1.70		
Number of people with chronic illness in household	0	73.90	71.40	9.85	0.197
	1	21.90	21.40		
	2	3.70	5.60		
	3	0.50	0.80		
	>3	0.00	0.40		
History of COVID-19	Had	2.70	14.40	65.45	0.000
	Had not	97.30	85.60		
History of COVID-19 in the household	Had	0.70	76.00	188.22	0.000
	Had not	99.30	24.00		

Note. None of the differences is significant. Households with more than 3 members were aggregated as they represented a very low percent.

### Main Analysis Results

#### Descriptive Statistics

The data of the two samples reflected typical Georgian households: very small percentages of both samples (7.3 and 8.7) lived alone, while the majority (30.5 and 32.8) shared a household with more than three individuals. In addition, more than 40% in each sample had at least one child and 30% had at least one elderly person in their households (see Table 1 above).

Furthermore, the big portion of both samples (48.3% in the first and 74% in the second wave) were worried about the health of family members and relatively few (11.2% in the first and 25.3% in the second wave) worried about own wellbeing. The worry about the economic consequences of the pandemic was a major concern in both samples (72% in the first wave and 71.5% in the second). In addition, while in the first sample a very small number of participants reported a personal (2.7%) or family (0.7%) history of coronavirus, in the second sample these numbers were significantly higher (14.4% and 24.0%, respectively with an 83.5% of overlap).

### Correlations

#### Main Variables

Data from the first wave showed that anxiety negatively correlated with life satisfaction and the action-planning coping style, and positively correlated with COVID-19 worry, regular and pandemic-specific coping behaviors and all other coping styles. COVID-19 worry was positively linked with all coping styles and coping behaviors, and negatively correlated with life satisfaction. Life satisfaction negatively corelated with both affective coping styles and positively correlated with both rational coping styles and pandemic-specific behaviors (see Table 2).

**TABLE 2:** Correlations among the variables (W1, over the diagonal and W2, below the diagonal) (Mental Well-being and Coping during the COVID-19 Pandemic, Georgia, 2020).

	1	2	3	4	5	6	7	8	9
1. Life satisfaction	1	−0.37**	−0.09*	0.04	0.10**	0.24**	0.09*	−0.13**	−0.22**
2. Anxiety	−0.40**	1	0.42**	0.11**	0.08*	−0.14**	0.13**	0.28**	0.50**
3. COVID-19 worry	0.04	0.36**	1	0.31**	0.28**	0.17**	0.38**	0.24**	0.32**
4. Regular behaviors	0.02	0.09*	0.36**	1	0.37**	0.19**	0.25**	0.05	0.06
5. Pandemic-Specific behaviors	0.04	0.14**	0.40**	0.54**	1	0.19**	0.15**	0.05	0.03
Coping styles									
6. Action-planning	0.31**	−0.33**	0.04	0.14**	0.09*	1	0.50**	0.06	−0.05
7. Information accessing/processing	0.14**	0.04	0.30**	0.25**	0.12**	0.45**	1	0.30**	0.30**
8. Passive-submissive	0.02	0.27**	0.23**	0.10**	0.10**	−0.07	0.17**	1	0.55**
9. Avoidant	−0.16**	0.48**	0.28**	0.15**	0.11**	−0.10**	0.25**	0.46**	1

Note. **p < 0.01; *p < 0.05.

In the second wave, anxiety positively correlated with COVID-19 worry, both emotion-focused coping styles and both coping behaviors, and negatively correlated with life satisfaction and the action-planning coping style. COVID-19 worry no longer correlated with action-planning coping style and life satisfaction, whereas coping behaviors positively correlated with anxiety, COVID-19 worry and all coping styles. Life satisfaction no longer correlated with passive-submissive style and pandemic-related coping behaviors, while anxiety no longer correlated with information-accessing/processing style. The newly emerged correlations in the second wave entailed positive links between both affective coping styles and both coping behaviors and a negative link between avoidant and action-planning coping styles (see Table 2).

### Demographic and Household Variables

In the first wave, weak correlations were established between: age and COVID-19 worry (*r* = 0.12, *p* < 0.01), age and information-accessing/processing style (*r* = 0.14, *p* < 0.01); number of children in the household and life satisfaction (*r* = 0.09, *p* < 0.05); number of elderly and pandemic-specific behaviours (*r* = 0.09, *p* < 0.01); number of family members with chronic illness, on one hand, and anxiety (*r* = 0.11, *p* < 0.01) and life satisfaction (*r* = −0.08, *p* < 0.05), on the other.

In the second wave, age still weakly correlated with life satisfaction (*r* = 0.11, *p* < 0.01), anxiety (*r* = −0.10, *p* < 0.01), COVID-19 worry (*r* = 0.14, *p* < 0.01), action-planning (*r* = 0.19, *p* < 0.01), information-accesing/processing (*r* = 0.18, *p* < 0.01) and passive sabmisive styles (*r* = 0.10, *p* < 0.01); number of children in a household positively correlated with action-planning coping (*r* = 0.08, *p* < 0.05) and information-accessing/processing style (*r* = 0.11, *p* < 0.01).

### Comparison of Means Between Two Waves

In total, nine variables–anxiety, COVID-19 worry, life satisfaction, four coping styles and two coping behaviors–were compared *via* MANOVA. According to Levene’s Test of Equality of Variances, five variables–COVID-19 worry, regular coping behaviors, avoidant, information-accessing/processing and action-planning coping styles–produced unequal variances; therefore, following Tabachnick and Fidell’s [36] advice, a stricter significance threshold of *p* < 0.01 was set up for them.

Ultimately, the means of six variables - anxiety, COVID-19 worry, life satisfaction, pandemic-related coping behaviors, the action-planning coping and the information-accessing/processing coping - markedly differed in two samples. Anxiety, COVID-19 worry and the information-accessing/processing style significantly increased, while life satisfaction, action-planning style and pandemic-related coping behaviors significantly decreased. The rise in the avoidant coping was notable, yet because of the stricter threshold did not meet the statistical significance criterion. The passive-submissive coping and regular coping behaviors generated insignificant differences (see Table 3).

**TABLE 3:** MANOVA results: between-group differences (Mental Well-being and Coping during the COVID-19 Pandemic, Georgia, 2020).

	Wave I (N = 750)		Wave II (N = 716)		*F*	*p*
	M	SD	M	SD		
Life Satisfaction	2.88	0.82	2.78	0.81	5.40	0.020
Anxiety	2.80	0.75	3.02	0.75	31.82	0.000
COVID-19 worry	2.76	0.96	3.35	0.94	143.89	0.000
Regular behaviors	4.04	0.77	4.05	0.86	0.00	0.960
Pandemic-Specific behaviors	4.17	0.88	4.07	0.95	4.19	0.041
Coping Styles						
Action-planning	3.49	0.74	3.38	0.86	7.45	0.006
Information-accessing/processing	3.09	0.76	3.22	0.85	10.20	0.001
Passive-Submissive	2.71	0.81	2.66	0.86	1.51	0.219
Avoidant	2.75	0.88	2.85	0.96	3.93	0.048

Note. Both significant and non-significant differences are provided. Life satisfaction was measured with The Satisfaction with Life Scale; Anxiety was measured with The State Anxiety Inventory; COVID-19 worry was measured with The COVID-19 Worry Scale; Regular and Pandemic-Specific behaviors were measured with The Pandemic-Related Coping Behaviors scale; Coping styles were measured with The Ways of Coping Scale.

As the personal or household history of COVID-19 of participants significantly increased during the second wave, we added these two variables as covariates into analysis and ran MANCOVA. As a result, four variables maintained significant differences between the sample means, while two–the information-processing coping style (*F* = 6.04, *p* > 0.01 no longer considered significant because of the stricter threshold) and pandemic-specific coping behaviors (*F* = 1.77, *p* > 0.05)—lost their significances. Thus, the levels of anxiety and COVID-19 worry increased, the level of life satisfaction decreased and so did the usage of the action-planning coping. Differences in pandemic-specific coping behaviors and the information-processing coping were no longer significant after controlling a personal/family history of coronavirus.

## Discussion

### Between-Wave Similarities and Differences

The descriptive statistics and the differences between the sample means of the waves revealed that the levels of anxiety and COVID-19 worry were below average in spring, 2020 and significantly increased by December 2020. While China and Western European countries suffered high prevalence of coronavirus and the associated high mortality in spring, Georgia had only 723 cases of infection and 12 deaths and reached high numbers in November–December 2020. Hence, the levels of anxiety and COVID-19 worry, and life satisfaction in both time periods corresponded to the threat coronavirus posed for the population. In line with our findings, other multiple-wave studies of the pandemic in different countries found the levels of anxiety, worry, and depression remaining present or rising over time [16, 17].

Next, our results showed that the action-planning coping and pandemic-specific coping behaviors significantly decreased over time. The avoidant coping also notably increased but not to the statistically signifiant degree. The worsening of mental health indicators (anxiety, COVID-19 worry, life satisfaction) and the reduction of action-planning and behavioral coping can be explained by the acute and prolonged collective stress caused by the pandemic and are consistent with the common adaptation syndrome [8] and findings of other studies [16, 17, 37]. The prolonged phase of stress may worsen public mental health and affect problem-oriented coping [38].

Nonetheless, the results indicated that individual or household contexts may shape the response to a collective stressor. Our findings suggested that, people with Covid-19 history tended to stop performing protective behaviors but continued seeking coronavirus-related information, while uninfected individuals tended to adhere to behavioural coping but were less inclined to seek information.

### Within-Wave Similarities and Differences

Correlational analyses of the two waves demonstrated that many links between the main variables remained constant over time: in particular, in both samples, anxiety positively correlationed with COVID-19 worry and both emotion-focused coping styles and negatively correlated with the action-planning coping style and life satisfaction. Similarly to our findings, anxiety was linked with emotion-focused coping in studies from Hungary and China [26, 39], and life satisfaction negatively correlated with perceived stress and avoidance, and positively correlated with problem-focused coping in an Italian study [40]. Yet, in the second wave, COVID-19 worry no longer correlated with the action-planning coping and life satisfaction, while life satisfaction lost its negative link with passive-submissive coping. These changes may suggest phase-specific variations in responding to the stressor.

### Main Findings and Implications

Our results may indicate that coping with acute and prolonged stress mostly implies the same basic scenarios; however, consistent with the common adaptation syndrome [8] and the transactional theory of stress and coping [9], they pinpointed that under the prolonged stress with an uncontrollable stressor, a worry caused by an actual threat may no longer be linked with task-oriented coping.

According to the transactional theory of stress and coping, the primary control (problem-focused coping) is advantageous when the stressor is controlable, while the secondary control (emotion-focused coping) is appropriate when the stressor is uncontrolable [9]. In line with this theory, at the acute stage of the pandemic, when the stressor was quite controllable considering the favorable epidemiological situation in Georgia in spring 2020, action-planning coping was higher and COVID-19 worry was strongly linked with problem-oriented coping, whereas by December 2020 along with the drastic rise in infection rates, the action-planning and behavioral coping significantly decreased, the avoidant coping exhibited the tendency to rise, and COVID-19 worry no longer correlated with the action-planning coping style. Similarly, a Polish study on coping with the second wave of the COVID-19 pandemic showed that acceptance and focusing on something else prevailed over problem-oriented coping [26].

The findings from our first wave linking rational fear of COVID-19 with better adaptation outcomes were also consistent with the Integrated Threat Theory (ITT), according to which perceived realistic threat predicts more rational, adaptive behaviors [41, 42]. During the prolonged stress phase, however, when the spread of infection rocketed making individuals feel less under control, the link between the threat-oriented worry and primary control weakened, suggesting that the adaptive function of rational fear might be time and resource-dependent.

Another valuable study finding is that the information-accessing/processing coping style significantly increased over time among those who contracted the virus, while uninfected Georgians were less inclined to cope *via* seeking information. These results highlight the need for informational support for people affected by COVID-19 and may also pinpoint the dominance of distressing pandemic-related news in media prompting uninfected individuals to avoid information. Thus, our findings suggested that continuous supply of targeted information at every stage of the pandemic is pivotal.

The main study limitations include its cross-sectional design and sampling bias. Men, older adults and individuals with low tech skills were underrepresented. Besides, common limitations of self-report surveys apply including the difficulty of calculating the response bias. Not measuring the level of stress may also constitute a limitation. Examining two large samples during the only two countrywide lockdowns is the key strength. Exploring moderation/mediational relationships between the variables may assist in better understanding how populations respond to global threats.

To sum up, the study findings demonstrated variations in emotional responses and coping of adult population during the acute and prolonged phases of collective stress, underlining the importance of timing, general context and the context of an individual in dealing with the global stressor. The findings pinpoint the potential burden of the pandemic on populations’ mental health and can be useful for both practice-based professionals and policy-level decision-makers.

### Conclusion

Comparisons of the data of the two waves from two lockdowns, one in the acute stress phase when the spread of the disease was very low and the other in the prolonged phase when the spread of the disease was very high, allowed to demonstrate that the adult population’s mental health deteriorated in the conditions of prolonged stress and their coping repertoire changed with a notable decrease in action-planning and behavioral coping and an increase in the information-accessing/processing style. It also showed that rational worry, linked with action-planning coping in the acute phase, no longer acted so during the prolonged phase of the pandemic. The findings allowed differentiating between the general countrywide context and the specific individual context of the stressful environment. While the general context was accountable for worsening mental health indicators and a decrease in the action-planning coping style, the specific context (household history of COVID-19) explained lower utilization of protective behaviors and higher information seeking. These findings highlight the importance of timing and context—both macro and micro—in coping with the global stressor and inform on measures for promoting population resilience.
